# Reviewers

**DOI:** 10.1111/jdi.12578

**Published:** 2016-11-01

The publication of invaluable papers in the Journal of Diabetes Investigation depends on the prompt, careful review of submitted manuscripts. We would like to thank the Editorial Board members, the International Editorial Board members, the Assistant Editorial Board members and the following experts for reviewing manuscripts from September 1, 2015 to August 31, 2016.

Toru Aizawa

Hitoshi Ando

Keiko Arai

Atsushi Araki

Shin‐ichi Araki

Syunnichi Ariizumi

Noriko Asahara

Shunichiro Asahara

Tomoichiro Asano

Yoshimasa Aso

Takuya Awata

Tetsuya Babazono

Anzel Bahadir

Preethi Balan

Sophie Beliard

Xiaofang Bian

Phillip Brantley

Juliana Chan

Te‐Fu Chan

Ting Fung Chan

Chee‐Jen Chang

Jer‐Ming Chang

Cheng‐Hsu Chen

Harn‐Shen Chen

Jin‐Shuen Chen

Liming Chen

Szu‐Tah Chen

Dong Hyeok Cho

Chih‐Hsun Chu

I‐Hua Chu

Hung‐Yi Chuang

Daisuke Chujo

I Cukić

Matthew D'Mello

Makoto Daimon

Takahisa Deguchi

Toshio Doi

Hengjin Dong

Masanori Emoto

Senneville Eric

Ulf Erikson

Satoshi Fujii

Kei Fujimoto

Shimpei Fujimoto

Wilfred Fujimoto

Shiho Fujisaka

Tomomi Fujisawa

Yukihiro Fujita

Yoshio Fujitani

Michiaki Fukui

Nobuaki Funahashi

Kengo Furuichi

Shinya Furukawa

Hiroto Furuta

Atsushi Goto

Weijun Gu

Honglei Guo

Yusuke Hada

Jong Ryeal Hahm

Yoshiyuki Hamamoto

Kumiko Hamano

Seung Jin Han

Kazuo Hara

Mariko Harada‐Shiba

F Haseda

Naotake Hashimoto

Mitsuru Hashiramoto

Tatsuya Hayashi

Tomoshige Hayashi

Jordi Heijman

Tsutomu Hirano

Takumi Hirata

Takahisa Hirose

Yushi Hirota

Kerry Hitos

Osamu Hori

Yukio Horikawa

Masaaki Hoshiga

Masayuki Hosoi

An‐Tsz Hsieh

Chang‐Hsun Hsieh

Hui‐Min Hsieh

Cheng Hu

Jee‐Fu Huang

Xiaoxu Huo

Kyu Yeon Hur

Chii‐Min Hwu

İlkay İdilman

Noriho Iida

Fotios Iliadis

Junta Imai

Fumiaki Imamura

Yasuya Inden

Toyoshi Inoguchi

Kazuo Inoue

Manami Inoue

Tatsuhide Inoue

Kouichi Inukai

Taiga Irako

Shun Ishibashi

Hisamitsu Ishihara

Hideki Ishii

Hitoshi Ishii

San‐e Ishikawa

Masanori Iwase

Jiann‐Shing Jeng

Mugdha Joglekar

Mikiko Kamijo

Hideki Kamiya

Kyuzi Kamoi

Keizo Kanasaki

Akio Kanazawa

Ippei Kanazawa

Hideaki Kaneto

Naoto Katakami

Ken Kato

Koichi Kato

Takumi Kawaguchi

Mitsunobu Kawamura

Takahiko Kawamura

Tomoyuki Kawamura

Daiji Kawanami

Eiji Kawasaki

Ryo Kawasaki

Jacob Keaton

Eric YH Khoo

Yoshiaki Kido

Bo Hyun Kim

Bu Kyung Kim

Chong Hwa Kim

Chul‐Hee Kim

Jae Hyeon Kim

Tadahiro Kitamura

Shigehiko Kitano

Seung‐Hyun Ko

Junji Kobayashi

Mitsuhisa Komatsu

Toshiyuki Komiya

Bokyung Koo

Daisuke Koya

Hidenori Koyama

Akira Kubota

Naoto Kubota

K Vijaya Kumar

Shoen Kume

Akio Kuroda

Takeshi Kurose

Selda Sayin Kutlu

Soo‐Heon Kwak

Hyuk‐Sang Kwon

Ismail Laher

Ting‐I Lee

Eun Young Lee

I‐Te Lee

Mei‐Yueh Lee

Hung‐Yuan Li

Weidong Li

Chia‐Huei Lin

Chun‐Liang Lin

Shu‐Fu Lin

Fang Liu

Shiro Maeda

Hiroshi Maegawa

Hideichi Makino

Yuichi Makino

Shoichi Maruyama

Hiroaki Masuzaki

Masafumi Matsuda

Munehide Matsuhisa

Eiji Matsuura

Aidan Mcelduff

Reinhold J Medina

Otsuki Michio

Silvia Migliaccio

Junnosuke Miura

Tetsuji Miura

Jun‐ichiro Miyagawa

Takeshi MIyatsuka

Hideaki Miyoshi

Tetsuya Mizoue

Hiroki Mizukami

Yasumichi Mori

Tatsumi Moriya

Masaaki Muramatsu

Yuko Murase‐Mishiba

Seiho Nagafuchi

Yoshio Nagai

Shoichiro Nagasaka

Kazuaki Nagashima

Tomoko Nakagami

Atsushi Nakagawa

Akinobu Nakamura

Nobuhisa Nakamura

Shuhei Nakanishi

Koh Nakayama

Takuma Narita

Masahiro Nishi

Rimei Nishimura

Takeshi Nishimura

Yoshihiko Nishio

Takashi Nomiyama

Shosaku Nomura

Hiroshi Noto

Atsushi Obata

Susumu Ogawa

Ken Ohashi

Mitsuru Ohsugi

Yoichi Oikawa

Yosuke Okada

Shiki Okamoto

Hiroaki Okazaki

Kwok Leung Ong

Hiroshi Onuma

Haruhiko Osawa

Seiichi Oyadomari

Nobuaki Ozaki

Priyadarshini Pantham

Giuseppe Papa

Cheol Whee Park

Kyong Soo Park

Yong‐Moon Park

Bengt Persson

Yong‐fen Qi

Venkatesan Radha

Sang Youl Rhee

Yoshifumi Saisho

Kazuhiko Sakaguchi

Hiroshi Sakura

Takashi Sakurai

Kazunori Sango

Hideyuki Sasaki

Toshiyasu Sasaoka

Jo Satoh

Ursula Schwab

Yusuke Seino

Osamu Sekine

Dinesh Selvarajah

Michael Shanik

Shiying Shao

YJ Sheen

Hirotaka Shibata

Kenichi Shikata

Akira Shimada

Masashi Shimoda

Iichiro Shimomura

Jun Shirakawa

D Simmons

Hitomi Suga

Takayoshi Suganami

Takashi Sugiyama

Shoichiro Sumi

Yasuharu Tabara

Yuji Tajiri

Hirokazu Takahashi

Kazuma Takahashi

Toshinari Takamura

Minoru Takemoto

Yoshikazu Tamori

Daisuke Tanaka

Kiyoshi Tanaka

Nagaaki Tanaka

Junichi Tani

Atsuhiro Tanikawa

Yasuo Terauchi

Haoming Tian

Masao Toyoda

Chin‐Hsiao Tseng

Chin‐Lin Tseng

Shoichiro Tsugane

Satoru Tsujii

Teresa Tusie‐Luna

Heiichiro Udono

Satoshi Ugi

Hiroyuki Umegaki

Seiji Umemoto

Toshitaka Umemura

Tatsuhiko Urakami

F Uygur

Takashi Uzu

Luca Valenti

Jun Wada

Hironori Waki

Jun‐Sing Wang

Shuangxi Wang

Jong Chul Won

Martin Wong

Chung‐Ze Wu

Jianzhong Xiao

Jing Xie

Kentaro Yamada

Satoru Yamada

Tetsuya Yamada

Tomohide Yamada

Yuichiro Yamada

Sho‐ichi Yamagishi

Ritsuko Yamamoto‐Honda

Yasuhiko Yamamoto

Shunsuke Yamane

Shwu‐Huey Yang

Yeoree Yang

Yutaka Yano

Hisafumi Yasuda

Hitoshi Yasuda

H Yilmaz

Koichi Yokono

Koutaro Yokote

Shin Yonemitsu

Kazuya Yonezawa

Narihito Yoshioka

Yukio Yuzawa

Miao Zhang

Sen Zhang

Shaoling Zhang

Xi Zhang

Hongting Zheng

Jian Zhou

Linuo Zhou

Xiaolin Zhu

